# Developmental time rather than local environment regulates the schedule of epithelial polarization in the zebrafish neural rod

**DOI:** 10.1186/1749-8104-8-5

**Published:** 2013-03-24

**Authors:** Gemma C Girdler, Claudio Araya, Xiaoyun Ren, Jonathan DW Clarke

**Affiliations:** 1 MRC Centre for Developmental Neurobiology, King’s College London, Guy’s Campus, London, SE1 1UL, UK; 2 MRC-LMB, Francis Crick Avenue, Cambridge Biomedical Campus, Cambridge, CB2 0QH, UK; 3 Laboratory of Developmental Biology, Institute of Marine Sciences and Limnology, Faculty of Sciences, Universidad Austral de Chile, Campus Isla Teja s/n, Valdivia, 5090000, Chile; 4 Invasive Pathogens Laboratory, Institute of Environmental Science and Research, 34 Kenepuru Drive, Porirua, Wellington, New Zealand

**Keywords:** Intrinsic program, Lumen, Neuroepithelial polarity, Neural tube, Zebrafish

## Abstract

**Background:**

Morphogenesis requires developmental processes to occur both at the right time and in the right place. During neural tube formation in the zebrafish embryo, the generation of the apical specializations of the lumen must occur in the center of the neural rod after the neural cells have undergone convergence, invagination and interdigitation across the midline. How this coordination is achieved is uncertain. One possibility is that environmental signaling at the midline of the neural rod controls the schedule of apical polarization. Alternatively, polarization could be regulated by a timing mechanism and then independent morphogenetic processes ensure the cells are in the correct spatial location.

**Results:**

Ectopic transplantation demonstrates the local environment of the neural midline is not required for neural cell polarization. Neural cells can self-organize into epithelial cysts in ectopic locations in the embryo and also in three-dimensional gel cultures. Heterochronic transplants demonstrate that the schedule of polarization and the specialized cell divisions characteristic of the neural rod are more strongly regulated by time than local environmental signals. The cells’ schedule for polarization is set prior to gastrulation, is stable through several rounds of cell division and appears independent of the morphogenetic movements of gastrulation and neurulation.

**Conclusions:**

Time rather than local environment regulates the schedule of epithelial polarization in zebrafish neural rod.

## Background

Morphogenesis is the process by which an organism takes its shape and it requires coordinated movements and shape changes of many cells and tissues. Furthermore, movement and shaping events must be coordinated with other fundamental processes of embryogenesis, such as proliferation, differentiation, and spatial patterning. Coordination through time ensures these processes occur in the correct sequence. Cells in different stages of development are likely to have different morphogenetic capabilities because processes such as differentiation of cells and tissues may limit the range of morphogenetic potential available to a cell. For example, mesenchymal cells will have a greater range of potential movements and rearrangements than epithelial cells, but epithelialization confers other possibilities for shaping tissues, such as folding. Although the mechanisms that regulate the sequence and timing of morphogenetic events are poorly understood, especially in vertebrates, it is well known that several different cell types are able to monitor developmental time. For example, somitogenesis is controlled by an oscillatory timer in the presomitic mesoderm (reviewed in [1]), and in the central nervous system, timers direct stereotypic programs of differentiation over time in retinal neuron precursors (reviewed in [2]), oligodendrocyte precursors (reviewed in [3]), and *Drosophila* neuroblasts [4]. Developmental timers can also initiate global transitions in development across the whole organism, controlling events such as the midblastula transition in *Xenopus*[5,6], the maternal to zygotic transition in zebrafish and *Drosophila* (reviewed in [7]), and the activation of an apoptosis program at gastrulation onset in *Xenopus*[8]. Moreover, the heterochronic gene network globally controls the timing and synchrony of cell-fate specification events across several different tissues in *Caenorhabditis elegans* development (reviewed in [9]).

One complex developmental process requiring the coordination of several cellular and molecular events in time and space is the morphogenesis of the zebrafish neural tube [10,11]. Here, the generation of a neural epithelium surrounding a central lumen involves the assembly of an apical surface within an initially solid neural rod primordium. Assembly of junctional complexes and polarity proteins at the midline marks the initiation of the columnar neuroepithelial architecture that is characteristic of vertebrate neural tubes. The emergence of neuroepithelial polarity is a critical step in this process that determines a distinct transition in cell behavior. Prior to this point, stable cell-cell junctions do not appear to be present within the core of the neural keel and this allows cells to undergo considerable rearrangements, including cell division and intercalation of cells across the midline of the keel and rod [11,12]. After epithelialization and lumen assembly, exchange of cells across the midline is not possible and a more stable epithelial structure is maintained by junctional belts that lie at the interface of apical and basolateral membrane domains and tether cells to their neighbors.

We and others have previously identified a novel and dominant influence of oriented cell divisions in organizing the developing lumen [11-14]. These C-divisions (for midline crossing divisions) occur close to the organ center, and during the C-division a green fluorescent protein fusion for the polarity protein partitioning defective 3 (Pard3-GFP) is often localized to the cleavage furrow between daughter cells [11]. Thus the C-division is normally coordinated spatially and temporally with apical polarization at the neural midline. This raised the possibility that the C-division itself could be responsible for localizing Pard3-GFP and related polarity proteins to the tissue midline. However by blocking the C-division we recently demonstrated that Pard3-GFP localization and assembly of apical specializations at the neural midline occurs independently of the C-division [10].

Two broad alternative mechanisms remain that might ensure the development of apical polarization at the correct time and place for lumen formation. One hypothesis is that cell extrinsic signals from the local environment determine the schedule of polarization. A second hypothesis suggests cells could begin polarization at a particular time in development while independent mechanisms regulate cell and tissue movements to ensure the polarizing cells are in the correct position. Evidence for this second scenario is suggested by the defects in lumen formation that arise when the convergence movements of the zebrafish neural plate are delayed either by compromised activity of the planar cell polarity pathway or surgical disruption of the neural plate midline [10,11]. In these embryos, apical polarization still occurs at approximately the right developmental time but no longer along the midline of the neural rod. This suggests the mechanisms that initiate cell polarization may be regulated by developmental time, but this remains to be tested experimentally.

In this work we have used a heterochronic transplant strategy to demonstrate that the development of apical polarization and the specific behaviors of the midline-crossing division are more strongly regulated by developmental time than local environmental signals. Furthermore we show this schedule of epithelial development is not dependent on a mechanism that counts cell divisions and is both independent of and resistant to environmental signals at the midline of the developing neural primordium.

## Results

### Neural cells polarize on time in ectopic locations in the embryo

**Figure 1:**
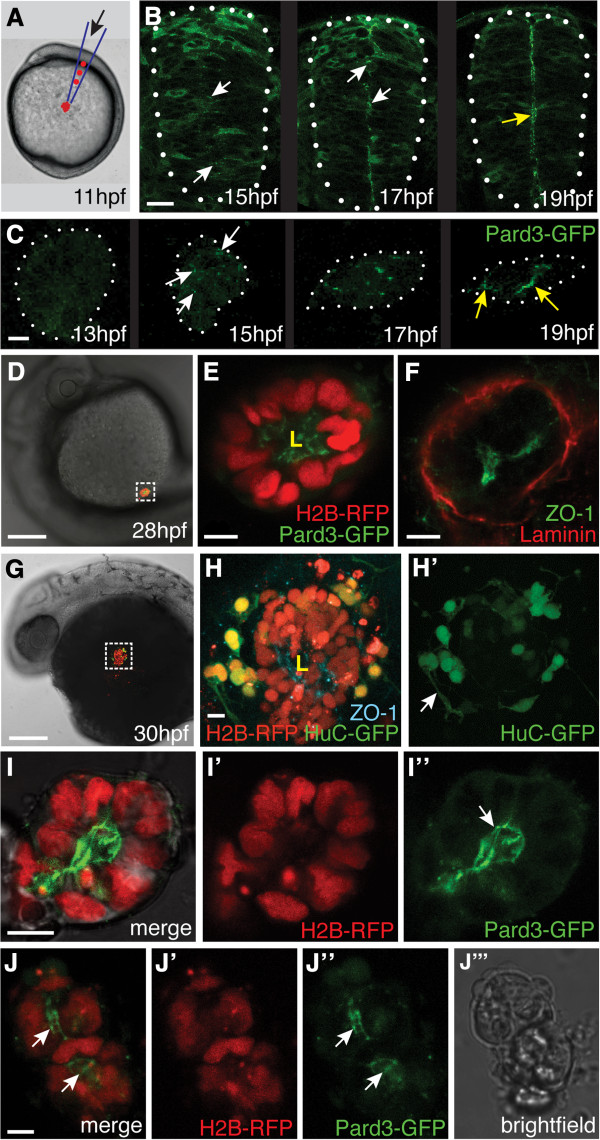
**Neural cells polarize on time in ectopic locations in the embryo. (A)** Schematic illustrating transplantation of 20 to 30 cells to the lateral surface of the yolk of a host embryo at 11 hours post fertilization (hpf). **(B)** Frames taken from a timelapse movie of a wild-type embryo showing the stages of green fluorescent protein/polarity protein partitioning defective 3 fusion (Pard3-GFP) polarization in the neural rod in transverse view (white dots outline the rod). White arrows indicate puncta of Pard3-GFP, yellow arrow indicates the apical surface expression of Pard3-GFP at the midline. Scale bar is 25 μm. **(C)** Maximum confocal projections of an ectopic cluster of cells from a timelapse movie showing similar timing of Pard3-GFP polarization to the neural rod (white dots outline the cluster). White arrows indicate puncta of Pard3-GFP, yellow arrows indicate strong Pard3-GFP coalescence at the cluster center. Scale bar is 10 μm. See also Additional file 1: Movie S1. **(D)** Ectopically transplanted cell clusters remain in an ectopic location at 28 hpf. Scale bar is 100 μm. **(E)** Magnification of the boxed region in D shows Pard3-GFP (green) is polarized to the center of cluster and marks a single lumen (L). Scale bar is 10 μm. **(F)** Zonula occludens 1 (ZO-1) (green) is located at the center of ectopic cluster outlining the lumen surface. The basal lamina component laminin (red) surrounds the cluster. Scale bar is 10 μm. **(G)** Ectopically transplanted cell cluster at 30 hpf. Scale bar is 100 μm. **(H)** Magnification of the boxed region in G. Neurons labeled with HuC-GFP (green) are present at the edge of the cluster and ZO-1 (cyan) demarcates a lumen (L) at the center of cluster. Arrow in **(H’)** points to neuronal processes. Scale bar is 10 μm. **(I)** A projection of z-slices though the center of a cluster of neuroepithelial cells expressing histone H2B/red fluorescent protein fusion (H2B-RFP) **(I’)** and Pard3-GFP **(I”)** cultured in Matrigel until 28 hpf. Pard3-GFP outlines a single central lumen (arrow in (I”)). **(J-J”)** A projection of z-slices though the center of a second cluster of neuroepithelial cells in Matrigel expressing H2B-RFP and Pard3-GFP with two lumens (white arrows). Scale bar indicates 10 μm. **(J”’)** Bright-field image of the cluster in (**J**).

We tested if the local environment of the neural rod is essential for cell polarization by removing neural progenitors from the neural plate and transplanting them onto the yolk under the enveloping layer (EVL) in a lateral location (Figure 1A). We found that by 28 hpf, ectopically transplanted cells were clustered together with Pard3-GFP localized to the center of the cluster outlining a small lumen (Figure 1D,E). Immunohistochemistry revealed that zonula occludens 1 (ZO-1) was localized to the center of the cluster indicating the presence of apical epithelial junctions, and the extracellular matrix component laminin surrounded the cluster at the cells’ basal surface (Figure 1F). The arrangement and polarity of cells within the cluster is similar to the structure of the neural tube and suggests that in these ectopic locations, the cluster of neural cells polarize and assemble a neuroepithelium that surrounds a central lumen. Timelapse imaging of these ectopic cells between 13 hpf to 22 hpf showed that Pard3-GFP became polarized within the ectopic cluster with the same timing as in the neural rod during neurulation (Figure 1B,C and Additional file 1: Movie S1). The specific environment of the embryo’s dorsal midline is therefore not required for neural cell polarization.

To verify that these ectopically located cells remain fated as neural, and to investigate if the differentiation program of these cells would continue in ectopic locations, we used donor neural plate cells from the tg(HuC-GFP) line, in which all neurons express GFP [15]. The neural tube of a 28 hpf wild-type embryo is organized with neurons differentiating at the basal edge and ZO-1 immunoreactivity lining the central lumen [16]. Following ectopic transplantation, cells at the periphery of clusters expressed HuC-GFP as early as 24 hpf. By 30 hpf, six out of the seven ectopic clusters examined contained HuC-GFP-positive neurons at the periphery of the cluster (Figure 1G,H). Moreover, some of the neurons had extended processes resembling axons or dendrites (Figure 1H’). Overall these experiments show that when transplanted into ectopic locations, clusters of neural cells not only polarize and generate lumens, but can also differentiate into neurons that exhibit typical neuronal morphology at the basal edge of the ectopic clusters.

### Neural progenitors form polarized cysts in three-dimensional gel cultures

To test if the neural cells’ potential to form epithelia is robust we investigated if they still retained this ability *in vitro* by embedding the neural plate cells in a three-dimensional Matrigel environment. Cells survived well within the matrix and by 28 hpf they had formed small epithelial cysts (Figure 1I-J”’). The shape of the cysts could be spherical (Figure 1I) or more irregular (Figure 1J). In the spherical clusters, Pard3-GFP was localized to the center of the cluster and lined a single lumen space (Figure 1I’). Larger, more irregular cysts could contain more than one lumen lined with Pard3-GFP (Figure 1J”). These results suggest neural plate cells have a self-organizing potential to generate epithelial ‘tubes’ that does not depend on the normal morphogenetic movements of the neural plate or the specific environment of the embryo’s dorsal midline.

### Heterochronic transplanted cells polarize according to their own schedule rather than the host schedule

**Figure 2:**
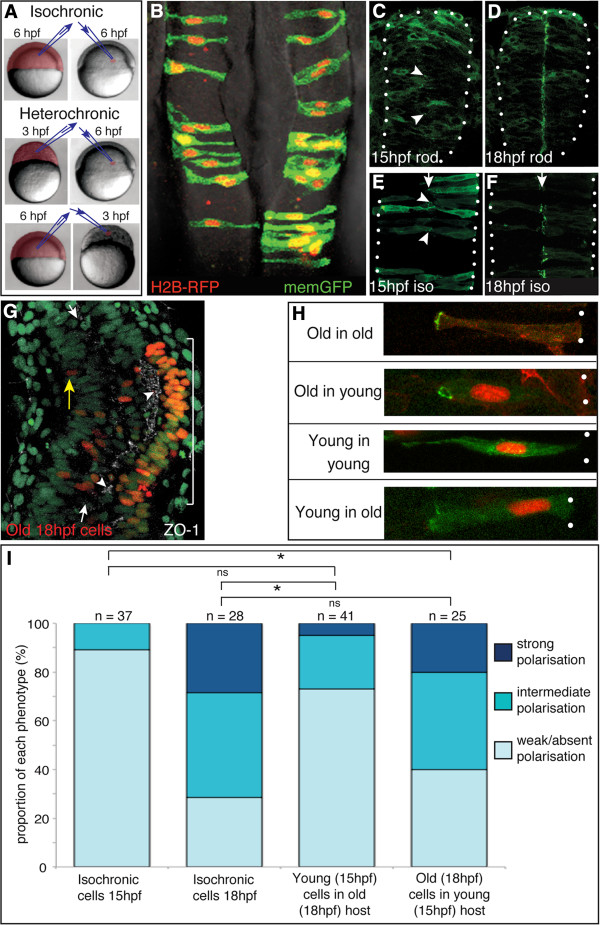
**Heterochronic transplanted cells polarize according to their own age rather than the host schedule. (A)** Schematic of isochronic and heterochronic cell transplantation strategy. **(B)** Isochronic transplanted cells integrate into the host neural tube and display the typical morphology of neuroepithelial cells at 24 hours post fertilization (hpf). **(C)** Green fluorescent protein/polarity protein partitioning defective 3 fusion (Pard3-GFP) is largely cytoplasmic in non-transplanted neural rod cells at 15 hpf, although there are a few small puncta at the developing midline (arrowheads). **(D)** Pard3-GFP is localized to the midline in non-transplanted neural rod cells at 18 hpf. **(E)** Isochronic transplanted cells at 15 hpf show Pard3-GFP localization typical of their age (compare to **(C)**), with some small puncta of Pard3-GFP (arrowheads). **(F)** Isochronic transplanted cells at 18 hpf show strong midline Pard3-GFP polarization, typical of their age (compare to **(D)**). **(G)** Dorsal view of 15 hpf host embryo (green nuclei) containing old (18 hpf) cells (red nuclei). Tissue has been stained for zonula occludens 1 (ZO-1) immunoreactivity (white) revealing high levels of ZO-1 expression adjacent to old cells (bracket) and low levels in regions containing only host cells. White arrows indicate neural midline. Yellow arrow indicates an isolated donor nucleus. **(H)** Pard3-GFP expression in isolated cells, transplanted isochronically (old into old and young into young) or heterochronically (old into young and young into old). White dots indicate basal surface. Scale bar 20 μm. **(I)** Quantification of Pard3-GFP polarization in isochronic and heterochronic cells. n, number of cells; ns, not significant; *P <0.01, Fishers test.

Our ectopic transplant experiments show the specific environment of the neural rod is not required to initiate neuroepithelial polarity. To test the alternative hypothesis that cells epithelialize at a particular time in development we investigated if heterochronic transplanted cells polarized according to their own age rather than the age of the surrounding host cells. Heterochronic cell transplantation was carried out at blastula stages, when 10 to 30 cells were transferred from donor embryos that were 3 h older or younger than host embryos. Cells were transplanted into the prospective hindbrain territory of host embryos (Figure 2A). Donor cells were labeled with Pard3-GFP to assess their apicobasal organization within the host neuroepithelium. We first analyzed the behavior of isochronically transplanted cells. These cells integrated well into the host neural tube. They extended fully across the apicobasal width of the neuroepithelium, dispersed among host neuroepithelial cells and established a spindle shaped morphology that is typical of neuroepithelial cells at 24 hpf (Figure 2B). Timelapse imaging of the transplanted cells during neurulation confirmed that they divided close to the midline at neural keel and rod stages and that the medial daughter cell crossed the midline to the contralateral side of the neural rod (Additional file 2: Movie S2). During normal neural tube development, Pard3-GFP is not strongly polarized in neural rod cells at 15 hpf (Figure 2C), but by 18 hpf Pard3-GFP is distinctly localized to the midline of the rod (Figure 2D). This timing of Pard3-GFP polarization is unaffected by isochronic transplantation (Figure 2E,F). Therefore isochronic cells show normal progenitor cell behaviors in the developing neural primordium.

In contrast to isochronically transplanted cells that dispersed fairly evenly in the host neuroepithelium, many heterochronically transplanted cells were found clustered together (bracket in Figure 2G). However, in addition to the clusters, most heterochronic transplants also contained some more isolated, outlying transplanted cells (yellow arrow in Figure 2G). To test the influence of host environment on the polarization of the transplanted cells we assessed polarization in isolated cells (Figure 2H) or cell pairs (rather than larger cell clusters), as these isolated cells will have had the maximal exposure to surrounding host cells. The level of Pard3-GFP polarization at the medial pole of donor cells was assessed ‘blind’ and categorized as strong, intermediate or weak/absent. We reasoned that if the timing of polarization depends on a cell’s age, transplanted older cells should show polarized Pard3-GFP when the young (15 hpf) host remains unpolarized. This is exactly what we saw, as many old in young transplanted cells had strong Pard3-GFP at the midline similar to old in old isochronic cells of the same age (Figure 2H). Statistical analysis of the level of Pard3-GFP polarization confirmed this result as we found no significant difference in the polarization of older transplanted (18 hpf) cells in a young host compared to isochronic transplanted cells at 18 hpf (*P* = 0.65 Fishers test, Figure 2I). Moreover, the polarization of older 18 hpf transplanted cells in a young host was significantly advanced compared to isochronic transplanted cells at 15 hpf (*P* <0.001 Fishers test, Figure 2I).

We next investigated if younger transplanted cells also polarized according to their age, and found that the large majority of young in old transplanted cells remained weakly polarized similar to isochronic young in young transplanted cells (Figure 2H). We found no significant difference in the polarization of young cells in older hosts or isochronic hosts (*P* = 0.13 Fishers test, Figure 2I) confirming that most younger cells show a level of polarization typical of their age rather than their environment. In agreement with the results above, we found that young 15 hpf transplanted cells in an old host were significantly less polarized compared to isochronic transplanted cells at 18 hpf (*P* <0.001 Fishers test, Figure 2I). We note that 5% of young transplanted cells localize Pard3-GFP in advance of their intrinsic schedule, but this small number indicates that environmental cues may only have a weak influence on this behavior.

In addition to monitoring the expression of Pard3-GFP in heterochronic cells we also assayed polarization using ZO-1 immunohistochemistry (Figure 2G). This had the advantage of revealing polarization in both host and transplanted cells, but the disadvantage that assigning polarization levels to individual cells was less certain. Nevertheless we found that ZO-1 was more strongly expressed and polarized adjacent to the older transplanted cells (arrowheads in Figure 2G), compared to the surrounding host tissue. This supports the Pard3-GFP result that older transplanted cells polarize prematurely compared to their surrounding host tissue, and is consistent with the hypothesis that the timing of polarization is more strongly regulated by the cells’ developmental program than by environment.

### Time rather than environmental influences regulate the mode of division at the neural midline

**Figure 3:**
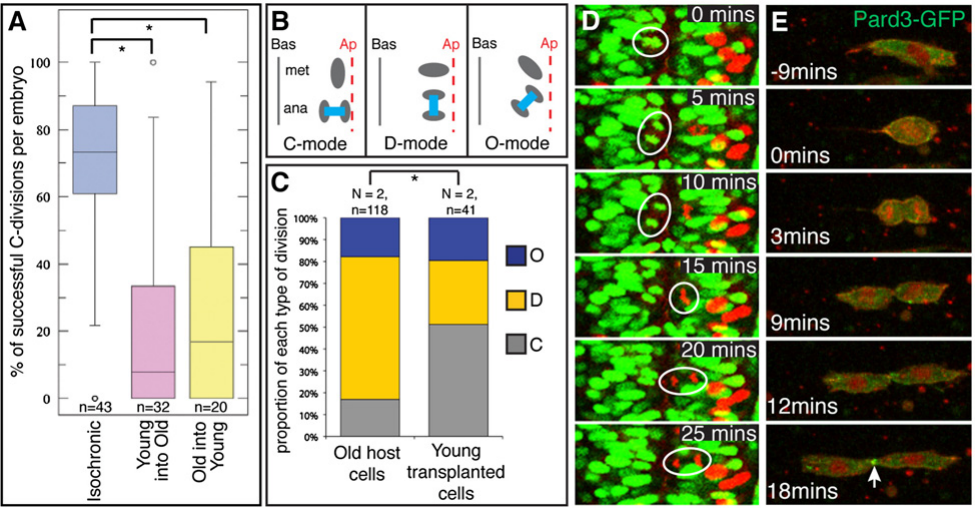
**The mode of cell division is regulated by intrinsic programs, not environmental influences. (A)** Quantification of midline crossing for isochronic and heterochronic cells; *P <0.01, KS test. n = number of embryos. **(B)** Schematic illustrating the three different modes of cell division orientation that occur at neural rod and tube stages. **(C)** Quantification of the mode of cell divisions for older host cells and younger transplanted cells. A significantly greater proportion of younger transplanted cells divide with a C-mode of division orientation compared to older host cells; *P <0.01, χ^2^ test. **(D)** Series of frames from a timelapse movie to illustrate a young cell (circled red nucleus) dividing with a C-division orientation, and a neighboring host cell (circled green nucleus) dividing with a D-division orientation. See also Additional file 3: Movie S3. **(E)** Sequence of timelapse frames of an old cell expressing green fluorescent protein/polarity protein partitioning defective 3 fusion (Pard3-GFP) transplanted into young host. During cytokinesis Pard3-GFP is localized to the abscission plane (arrowed) as is typical of cells at neural rod stages but not at earlier stages of development.

Prior to epithelialization most cells in the neural rod undergo an oriented division (called the C-division) close the neural midline. The orientation of the C-division and its location at the neural midline usually results in one of the two daughters crossing the midline to the other side of the neural rod [11,17]. If the C-division is regulated by the local environment of the neural midline then heterochronically transplanted cells would be expected to undergo this cell behavior when they reach the midline. To get an approximate assay of this behavior we quantified the number of cells that had crossed the midline following unilateral transplants of heterochronic or isochronic cells. We found that in the large majority of both young into old and old into young heterochronic transplants cells were unable to generate a midline crossing event. This was significantly different to isochronically transplanted cells that crossed the midline with a mean efficiency close to 80% (Figure 3A). This result suggests the local environment is insufficient to promote the midline crossing division in heterochronically transplanted cells.

To assay more precisely the roles of environment and time in regulating the orientation of the C-division we have combined heterochronic transplantation with timelapse microscopy. The cleavage plane of the C-division is oriented parallel to the midline plane [13,14,18]. However in the very next division of neural cells (which we will call the D-division) the cleavage orientation changes by 90°, so that daughter cells separate along an axis parallel with the developing ventricular surface [16]. We therefore wanted to test whether these distinct modes of division are also regulated by age rather than environment. To test this we monitored divisions of host and heterochronically transplanted cells. We quantified division orientation after young cells were transplanted to an older host and classified cell divisions as either C-mode or D-mode, or as O-mode divisions if the separation of daughters was oriented at an oblique angle to the midline (Figure 3B). We found that at a time when the majority of host cells are dividing in the D-mode the majority of the adjacent young transplanted cells maintained their C-mode of division (Figure 3C,D and Additional file 3: Movie S3). Isolated heterochronically transplanted cells are able to maintain their own program of division orientation despite being surrounded by host cells of a different age (Figure 3D), thus the orientation of division of most cells appears to be regulated according to their age rather than their environment.

Another characteristic of cells that divide across the midline in the neural rod is that they localize the Pard3-GFP fusion protein to either their cleavage furrow or their abscission plane [11]. Although it is difficult to predict when heterochronically transplanted cells will divide we have managed to analyze two such divisions by timelapse microscopy. Both cases were of older cells transplanted to a young host. Both cells divided when the host was approximately 13 hpf old and localized Pard3-GFP to the abscission plane as they completed cytokinesis (Figure 3E). This observation is consistent with the older cells maintaining their own developmental program of division and polarization, however although this behavior is most often seen in neural rods older than 13 hpf a few host cells at 13 hpf may also be expected to divide in this manner.

### Neural progenitors do not count the number of cell cycles to regulate polarization and mode of division

**Figure 4:**
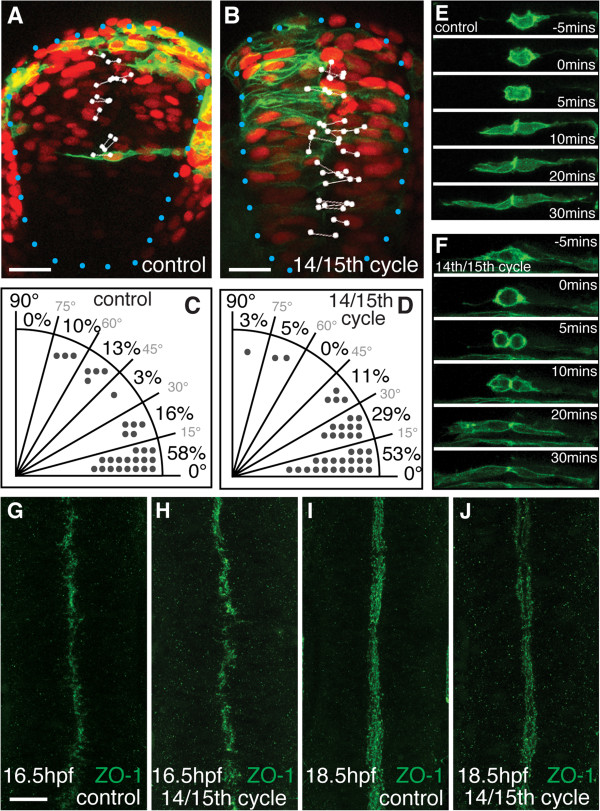
**Neural progenitors do not count the number of cell cycles to measure time. (A,B)** Most 16th cycle cell divisions in control embryos occur close to the midline with a mediolateral orientation. In a 14th/15th cycle embryo, most cells in the neural rod still divide close to the midline with a mediolateral orientation. The larger nuclei in the 14th/15th cycle embryos confirm that cells have undergone fewer divisions than controls. White dots indicate location of anaphase nuclei and the lines indicate division orientation. Blue dots outline edge of neural rod. Scale bar is 25 μm. **(C,D)** Quantification of cell division orientation in the neural rod of control (**(C)**, n = 29) and 14th/15th cycle (**(D)**, n = 38) embryos. An angle of 0° represents a mediolateral separation of daughter cell nuclei. An angle of 90° represents dorsoventral oriented separation of daughter cell nuclei during mitosis. **(E,F)** Timelapse frames from a control **(E)** or 14th/15th cycle **(D)** embryo. For both divisions, the cell rounds up and divides with a mediolateral orientation close to the midline at t = 0, and sister cells then separate across the midline by t = 30 minutes. **(G-J)** Dorsal view confocal projections through the hindbrain of zonula occludens 1 (ZO-1) staining in control or 14th/15th cycle embryos. At 16.5 hours post fertilization (hpf), ZO-1 is localized to the midline of the neural rod in control **(G)** and 14th/15th cycle **(H)** embryos. At 18.5 hpf, ZO-1 outlines the apical surface of the neural tube, and the ventricle has begun to open both in control **(I)** embryos and 14th/15th cycle **(J)** embryos. Scale bar is 25 μm in all figures.

Our heterochronic and ectopic transplant experiments suggest the development of apical polarization in neural cells is more strongly regulated by developmental time than local environment. To begin to address the question of how cells may monitor time we have tested the hypothesis that they count cell divisions as a way of measuring elapsed time. Cell divisions and cell cycle times are rather stereotyped in the zebrafish embryo and this means distinct cell behaviors often occur during a particular cell cycle. For example the midline crossing division is almost always a cell’s 16th cell division [17]. Since this division is often coincident with the appearance of apical polarity [11] it is conceivable that progression through a certain number of cell cycles could be one method that cells use to determine developmental time. To test this we temporarily inhibited cell division prior to neurulation from 6 hpf to approximately 11 hpf using the pharmacological inhibitors aphidicolin and hydroxyurea (Additional file 4: Figure S1, [11,12,19-21]). This is predicted to block one or two rounds of cell division [17]. Thus, upon washout of the inhibitors and subsequent recovery of cell division, cells in these embryos should be undergoing either their 14th or 15th cell division instead of their 16th. Despite missing one or two rounds of division, morphogenesis of the neural rod occurs on schedule. Simultaneous timelapse imaging of experimental embryos alongside control embryos showed that many cells recover from the division block to undergo division at rod stage of neurulation (15 to 17 hpf; Additional file 5: Movie S4). Previous work shows cells undergoing their 14th and 15th cell division usually divide lateral to the midline and along the anteroposterior axis of the embryo, whereas cells undergoing their 16th division divide along the mediolateral axis and cross the midline [17]. However we found that the locations of most divisions in 14th/15th cycle embryos were close to the midline of the neural rod, similar to the location of cell divisions in control embryos (Figure 4A,B). We also found no significant difference in division orientation compared to control embryos (*P* >0.39, Kruskal-Wallis test, Figure 4C,D). Finally, as a defining characteristic of 16th cell division is the separation of sister cells across the midline, we also analyzed if cells crossed the midline after division in 14th/15th cycle embryos. We found that 85% of these divisions resulted in one daughter cell crossing the midline (Figure 4F and Additional file 5: Movie S4; n = 70, two embryos), and thus showed similar behaviors to 16th cycle divisions (Figure 4E). We conclude that most cell divisions in the neural rod of 14th/15th cycle embryos show the properties of normal 16th cycle cell divisions.

To directly assess whether apicobasal polarity is established as normal in these 14th/15th cycle embryos we monitored ZO-1 immunoreactivity. At 15 hpf, both control and 14th/15th cycle embryos showed only faint puncta of ZO-1 (data not shown). Similarly at 16.5 hpf and 18.5 hpf, there was no consistent difference in ZO-1 staining between control and 14th/15th cycle embryos (Figure 4G-J). This experiment demonstrates that at the tissue level, the establishment of apical polarity is not disrupted in 14th/15th cycle embryos. Together these experiments show neural cells do not count the number of cell divisions as a mechanism of monitoring time to determine when to polarize and undergo the midline crossing division.

### Heterochrony leads to morphogenetic defects in the neural tube

**Figure 5:**
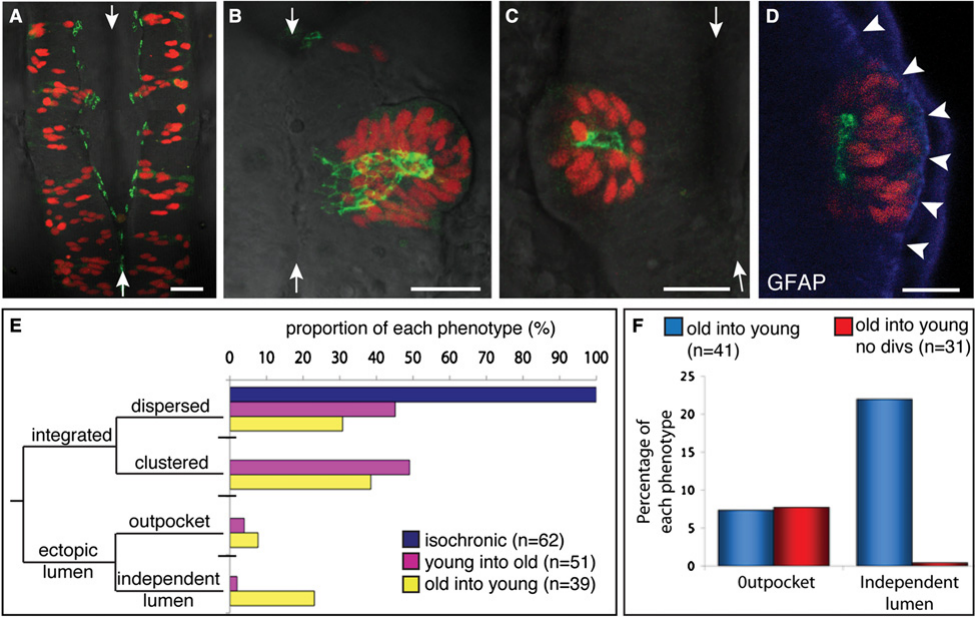
**Heterochronic transplanted cells integrate into the host neuroepithelium, and can generate ectopic lumen surfaces. (A-D)** Dorsal view projected confocal z-series of transplanted cells in the host hindbrain at 24 hours post fertilization (hpf). White arrows indicate the midline of neural tube. Scale bar is 25 μm. **(A)** By 24 hpf, isochronic transplanted cells have established apicobasal polarity, as revealed by green fluorescent protein/polarity protein partitioning defective 3 fusion (Pard3-GFP) localization at the ventricular surface of the neuroepithelium. **(B)** Heterochronic transplanted cells can generate ectopic apical surfaces that form an outpocket of the host neuroepithelium. (**C**) Heterochronic transplanted cells can form rosettes with a lumen independent of the host lumen. **(D)** Glial fibrillary acidic protein (GFAP) (blue) staining confirms that an ectopic rosette of transplanted cells is located within the neuroepithelium. Arrowheads show lateral edge of neural tube. **(E)** Frequency histogram showing the proportion of isochronic and heterochronic transplanted cells that fall into the four phenotypes of integrated dispersed, integrated clustered, ectopic lumen outpocket or independent lumen. **(F)** Graph showing that blocking cell division abolishes formation of independent lumens in old into young transplants.

To investigate whether uncoupling the schedule of neural development from other aspects of morphogenesis is detrimental to neural tube development, we analyzed the morphology of the neural lumens following heterochronic transplants. In comparison to isochronic transplanted cells that all integrate into the host neuroepithelium (Figure 5A) we found deformed (Figure 5B) and ectopic lumens (Figure 5C) were generated in 31% of old into young transplants, and 6% of young into old transplants (Figure 5E). The ectopic apical surfaces displayed a reticular pattern of Pard3-GFP (Figure 5B), which is characteristic of the network of junctional rings that colocalizes with ZO-1 protein at the apical ends of cells [16]. Wholemount immunohistochemistry for the basal marker glial fibrillary acidic protein (GFAP) confirmed that the transplanted cells generating ectopic lumens were still located within the host neuroepithelium, as GFAP staining was present around the outer edge of the cluster, continuous with the outer edge of the host neural tube (Figure 5D). As the formation of ectopic lumens by heterochronic transplanted cells occurred most often when older cells were transplanted cells into a young host, this suggests that groups of old cells self-organize more readily than young cells within the host tissue. Since we have previously shown that a midline-crossing division (the C-division) is an important organizing influence during neural lumen formation [11], we wondered if cell divisions play a role in the formation of ectopic lumens. To test this we blocked cell division over the period of neurulation and found that this treatment completely abolished the formation of independent lumens by older transplanted cells (Figure 5F). This shows that the ability of heterochronic cells to form independent lumens depends on cell division. It suggests the older transplanted cells undergo the specialized C-divisions precociously compared to host cells, and this can organize an ectopic apical surface in the center of the cluster of transplanted cells.

## Discussion

Embryogenesis must be organized both in time and space. While considerable effort has investigated the regulation of positional identity, comparatively little has been aimed at understanding how developmental events are coordinated through time. In our experimental system, the morphogenesis of a central lumen within the solid neural rod of the zebrafish embryo requires the development of apicobasal polarization of neural cells to be coordinated with the cell rearrangements such as intercalation and cell division that occur across the tissue midline [11]. Our current study aimed to improve our understanding of how this coordination of polarization and midline morphogenesis is achieved. In normal development, zebrafish neural cells do not develop overt neuroepithelial polarity (that is, become part of a coherent sheet of cells anchored with their basal ends adjacent to the basal lamina and with their apical ends characterized by a belt of junctional proteins at a distinct apicobasal interface) until the end of the neural rod stage. At that time the apical ends of cells are aligned along the midline plane of the neural rod and able to form an apical epithelial surface. This organization then allows the lumen to open along this midline plane. The neural rod stage is preceded by the neural plate and neural keel stages. In the neural plate, cells do not form an overt apical surface and do not appear to have polarized expression of apical polarity proteins such as Pard3, atypical protein kinase C (aPKC) and ZO-1. At neural keel stages, the cells are converging to the dorsal midline and invaginating to form a keel shaped primordium. During this phase the apical polarity proteins are detected progressively closer to the neural midline [11,18,22]. Although these observations and the development of overt apical epithelial polarity at the neural rod midline suggests apical polarity could be triggered by spatial cues at the midline, our current results show the local environment is not necessary for apical polarization, and in fact polarization is more strongly regulated by the age of the cells.

Our results show that heterochronic cells are able to retain their own temporal program from the time of transplantation at blastula stages (3 hpf) through to late neural keel stage (16 hpf), which indicates that the timer must operate from an early stage in development and be initiated even before cells are committed to a neural fate. Moreover, the schedule is robust enough to resist being reset by the many environmental signals and cell-cell interactions that occur after cell transplantation. The embryo not only undergoes extensive morphogenesis during the processes of epiboly, gastrulation and neurulation in this time, but cells also progress through four cell cycles, none of which appear to have significant affect on the cells’ original schedule of polarization.

The generation of neural cysts in ectopic locations in the embryo and in three-dimensional gel cultures shows that neural cells have a strong propensity to self-organize into a basic epithelium that surrounds a lumen. Rosettes that resemble epithelial organization around a lumen have also been reported in ectopic locations within the neural tube of Cdh2 mutant zebrafish [23]. This potential to generate cysts is also seen in other epithelial cell lines grown in culture (reviewed in [24]). However, the normal neural lumen forms from a rather precise midline plane (for example see Figure 1B), but in the ectopic neural cysts the lumen shape is much more variable. Thus although the program for epithelialization within neural cells may be regulated by time, it is very unlikely that time alone is sufficient to explain the precise spatial organization of apical junctions that are characteristic of the wild-type neural rod lumen. Once apical polarization is initiated according to the cells developmental schedule, we hypothesize that the assembly of apical junctions along the plane of the neural rod midline will be orchestrated by a combination of local cues and cell-cell interactions at the tissue midline. Local organizing cues are likely to include the basal lamina [10,25,26] as well as other polarizing or adhesive signals [13,14] and cell-cell interactions will include those where cells from the left and right hand sides meet at the midline [10].

In addition to apical polarization, another distinctive cell behavior usually occurs at or close to the midline of the neural rod (this is the midline-crossing division that generates mirror-symmetric daughters on either side of the neural midline) [11,17]. The mediolateral orientation of the midline-crossing division is distinct from the divisions that precede and follow it, both of which tend to be oriented parallel to the midline [16,17,27]. Our heterochronic transplants show the orientation of divisions at the midline is also more influenced by the transplanted cells’ age than their heterochronic environment. Since the orientation of midline-crossing divisions is regulated by Scribble [14] and the non-canonical Wnt receptor Frizzled 7 [13] this suggests these regulatory systems may also be sensitive to developmental time. It may be that many aspects of the cells’ differentiation program are sensitive to developmental time not just their epithelialization program. Therefore it may be instructive in the future to examine other aspects of their development such as the timing of cell fate induction and neuronal differentiation. Despite the fact that apical polarization and the characteristic properties of the C-division are closely coordinated in time and location in both normal and experimental conditions, our recent work demonstrates that apical polarization occurs independently of the C-division [10]. In addition our current work shows that cells do not monitor developmental time by counting progression through a certain number of cell cycles. Thus, neither progression through the C-division nor other rounds of division is part of the mechanism that allows neural cells to monitor developmental time.

One feature of heterochronically transplanted cells is their tendency to cluster together rather than disperse evenly among the host neural cells. This suggests the adhesive properties of cells change over time. Several lines of evidence suggest cell adhesion itself could be upstream of polarizing mechanisms such as microtubule organization and centrosome position [28,29], and thus may be important in setting up epithelialization and lumen formation [30,31]. These considerations suggest changes in adhesive properties may be one of the important cell characteristics to be regulated through developmental time.

### Possible mechanisms

At present we do not know the mechanism that allows cells to measure and respond to developmental time. Lineage studies in the zebrafish have correlated particular cell cycles with particular morphogenetic steps [17], suggesting neural cells may monitor time by counting cell cycles. Although cytokinesis is required in specific switches during the sequential generation of neurons by distinct transcription factors in the *Drosophila* nervous system [32,33], our experiments show that cells do not monitor time by counting the precise number of cell cycles. The zebrafish embryo must have a different way of measuring developmental time. A global mechanism similar to the heterochronic genes in *C. elegans*[9] may operate, in which integration of timing information with the extrinsic environment provides specificity of cell behavior in different tissues. Alternatively, an egg-timer-like mechanism may operate that consists of an intracellular factor that gradually increases or decreases in concentration or activity over time, similar to p27 and p57 that control the time of differentiation of optic nerve oligodendrocyte precursors *in vitro*[3]. In zebrafish microRNAs (miRNAs) might be attractive molecular candidates for the timer as they are important components of several intracellular timers. miRNAs are small 22 nucleotide RNAs that function to repress gene function, either by binding to their target mRNA and inhibiting translation, or by directing the cleavage of the target mRNA [34,35]. Not only are several heterochronic genes in *C. elegans* miRNAs [36-38], but miRNAs have also been implicated in retinal histogenesis [39] in which intrinsic timing mechanisms play an important role in several species [40-42]. Finally, the zebrafish MZ*dicer* mutant, that is unable to process precursor miRNAs, shows a range of morphogenetic defects including neural tube defects related to brain ventricle development [43].

### Morphogenetic advantage

For normal morphogenesis of the neural tube, the schedule that regulates apical polarization must be tightly coordinated with the mechanisms controlling convergence movements of the neural plate. When tissue movements and polarization become uncoupled, defects in neural tube morphogenesis arise (this study and [11]). These manipulations reveal an inherent fragility to this morphogenetic strategy and raise the question as to why the zebrafish embryo employs a timing strategy for initiating cell polarization and lumen formation? In most vertebrates a polarized neuroepithelial organization is established at the neural plate stage, which is much earlier in neural tube morphogenesis than in the fish. The fish may therefore use or adjust a timing mechanism to delay neural epithelialization beyond the neural plate stage. We suggest this delay is essential to enable the neural cells to undergo the complex behaviors of midline crossing divisions and cell intercalation during the keel and rod stages of fish neurulation. These cell behaviors may be especially advantageous to fish embryos whose neural tubes form along the curvature of the spherical yolk mass, as excessive curvature appears to be detrimental to neural tube closure in many vertebrate embryos [44-47]. Interdigitation and intercalation of cells across the neural midline in fish may ensure a mechanically stronger strategy for left-right fusion compared to epithelial folding and neural fold fusion in other vertebrates. A cell intrinsic timing mechanism that avoids neural polarization at the neural plate stage thus endows the fish cells with the potential for more sophisticated behaviors at the midline than other vertebrate neural plate cells are capable of. Although extensive left-right interdigitation has not been described during neural tube closure in other vertebrates, filopodial and lamellipodial protrusive activity is observed in the regions of left-right fusion [48-50]. This may indicate that some aspects of epithelial organization are modified during this fusion event in other vertebrates.

## Conclusions

Our series of ectopic transplants and heterochronic transplants have determined that the development of conventional neuroepithelial polarity in the zebrafish neural rod is more strongly regulated by developmental time rather than local environmental signals. In combination with other morphogenetic processes such as the planar cell polarity pathway that ensure the cells converge to the midline on time [11], this strategy ensures neural tube formation in the fish is a robust and efficient process.

## Methods

Embryos were raised according to standard protocols and staged in hours post fertilization (hpf) [51,52].

### mRNA injection and cell labeling

Embryos were labeled by injection of RNA encoding histone H2B/red fluorescent protein fusion (H2B-RFP) (100 pg), H2B-GFP (100 pg), membrane-GFP (memGFP, 100 pg), membrane-Cherry (100 pg) or Pard3-GFP (100 pg). Donor embryos for transplantation were injected at the 1 to 2 cell stage to ensure ubiquitous labeling. For mosaic labeling, RNA was injected into a single cell at the 16 to 64 cell stage.

### Heterochronic cell transplantation

For control isochronic transplants, embryos of the same age collected at the same time were used for donor and hosts. For heterochronic transplants, donor and host embryos were obtained with a 3-h time difference. All embryos were subject to the same temperature conditions until transplantation to maintain the original time difference between donors and hosts. Transplantations were carried out at blastula and gastrula stages (between 3 to 7 hpf) in agarose-lined dishes containing fish water supplemented with penicillin and streptomycin (Sigma, Gillingham, UK) [52-54]. Approximately 10 to 20 cells were extracted from the donor embryo using a micropipette and expelled into the host embryo in the prospective hindbrain region. Host embryos were unlabeled except for timelapse imaging of young cell divisions when the older host was ubiquitously labeled with H2B-GFP.

For cell polarization analyses, transplanted host embryos were fixed in 4% paraformaldehyde. Old hosts were fixed at 17.5 to 18 hpf when Pard3-GFP should be strongly polarized, and young hosts were fixed at 14.5 to 15 hpf when Pard3-GFP polarization should be weak or absent.

### Midline crossing analysis following heterochronic transplantation

For the midline crossing analysis of heterochronic cells, the location of transplanted cells was assessed at 10 to 11 hpf, and only unilateral distributions were imaged later. Midline crossing was used as an indirect measure of whether cells have undergone C-division at the normal time and place, and was assessed by counting the number of integrated cells on each side of the neural tube at 22 to 24 hpf. At this stage all transplanted cells should have undergone a C-division, and most cells should not have divided again. As all transplanted cells were initially unilateral, and cells are only able to cross the midline after division [11], the percentage of cells crossing the midline was calculated by dividing the number of cells on the contralateral side by the total number of cells × 100. Assuming that all cells will have attempted a C-division and produced two daughter cells, then doubling this percentage gives the percentage of successful C-divisions that have occurred in each embryo.

### Ectopic yolk transplants

Approximately 20 to 30 neural plate cells were taken from a labeled wild-type or HuC-GFP donor embryo at 11 hpf using a glass micropipette and immediately expelled just under the enveloping layer above the yolk in a central lateral location in an unlabeled host embryo. Host embryos were incubated overnight in fish water supplemented with penicillin and streptomycin until analysis at 28 to 30 hpf.

### Matrigel three-dimensional culture

For this experiment we used cells from the neural plates of MZ*oep* mutant embryos as their anterior neural plate is enlarged and it is easier to harvest large numbers of cells. These donor embryos were ubiquitously labeled with Pard3-GFP and H2B-RFP. When donor embryos were 11 hpf, a 30 μl drop of Matrigel culture medium (50% Matrigel Phenol Red-free (BD Biosciences, Oxford, UK), 39% L-15 media (Gibco, Life Technologies, Paisley, UK), 10% fetal calf serum, 1% penicillin/streptomycin (Sigma, Gillingham, UK)) was placed on a glass coverslip or petri dish on ice. Then 20 to 30 cells were removed from the donor neural plate using a capillary needle and micromanipulator and immediately but slowly expelled into the Matrigel drop. The Matrigel drops were left to gel for 30 to 60 minutes at room temperature, and then covered with a solution of 2% Matrigel in L15 (including 5% FCS and 1% 150 μl penicillin/streptomycin) and incubated at 28.5°C until imaging.

### Blocking cell division

To block cell division during neurulation, a combination of the cell division inhibitors aphidicolin and hydroxyurea was used [11,12,19-21]. First, both experimental and control embryos were injected at the 1 to 2 cell stage with 1 nl of 1 mM p53 morpholino (p53MO, 5’-GCGCCATTGCTTTGCAAGAATTG-3’, Genetools Inc., Philomath, OR, USA) to reduce cell death. Experimental embryos were then treated from 6 to 11 hpf with 150 μM aphidicolin (Enzo Lifesciences, Exeter, UK) and 20 mM hydroxyurea (Sigma, Gillingham, UK) in 2.5% dimethylsulfoxide (DMSO) in embryo medium to inhibit division for at least one cell cycle, and control embryos were incubated in 2.5% DMSO in embryo medium for the same time period.

### Immunohistochemistry

Embryos were fixed for 2 h at room temperature or overnight at 4°C. Primary antibodies used were anti-ZO-1 (1:500, Life Technologies, Paisley, UK), anti-GFAP (1:500, Z0344 Dako, Ely, UK), anti-phosphohistone H3 (1:500, Millipore, Billerica, MA, USA), and anti-laminin (1:500, L9393 Sigma, Gillingham, UK). Secondary antibodies were fluorescently conjugated anti-mouse, anti-rabbit or anti-chick alexa-488, alexa-546, alexa-633 or alexa-647 (1:400, Life Technologies, Paisley, UK). Embryos were counterstained with nuclear To-Pro or Sytox stains (1:1,000, T3605 or S7020, Life Technologies, Paisley, UK,) for 1 h after secondary antibody incubation when required.

### Confocal and timelapse imaging

Images were acquired on a SP5 Leica confocal microscope. Embryos were mounted in low-melting-point agarose and oriented for dorsal or transverse views of the neural primordium, or oriented laterally for imaging of ectopic transplanted cells. Z-slices were acquired with a z-interval of 2 to 5 μm. For timelapse imaging, a z-stack was captured every 5 to 7 minutes. When imaging embryos at 22 to 30 hpf, embryos were anaesthetized with MS222 (Sigma, Gillingham, UK) and treated with 0.003% 1-phenyl-2-thiourea (w/v) to prevent pigmentation. To analyze Pard3-GFP polarization of transplanted cells, all settings on the confocal were standardized and images were analyzed without subsequent processing by an independent investigator who was ‘blind’ to the embryos experimental status. Imaging of cells in Matrigel was carried out live, by dipping a water immersion lens into the L-15 medium.

### Statistical analysis

The Student’s t test was used to test for a significant difference between the percentages of successful C-divisions in isochronic transplants compared to division-blocked isochronic transplants, as the data were of a Gaussian distribution. The Kolmogorov-Smirnov (KS) test was used to test for a significant difference between the percentages of C-divisions in isochronic transplants and each type of heterochronic transplant because the data were of a skewed distribution. This test was performed online using the ‘Statistics to Use’ website (http://www.physics.csbsju.edu/stats/). The χ^2^ test (GraphPad Prism) was used to test for a significant difference between the cell division orientations of young transplanted and host cells from a 3 × 2 contingency table of C, D or oblique angled divisions for the two ages of cells.

Separate χ^2^ (GraphPad Prism) or Fishers tests were used to test for significant differences between the Pard3-GFP polarization state of heterochronic transplanted cells compared to isochronic transplanted cells at 15 hpf and 18 hpf.

The Kruskal-Wallis test (GraphPad Prism) was used to test for a significance difference between the orientation of cell divisions in control and 14th/15th cycle embryos.

## Abbreviations

aPKC: Atypical protein kinase C; GFAP: Glial fibrillary acidic protein; hpf: Hours post fertilization; ZO-1: Zonula occludens 1.

## Competing interests

The authors declare that they have no competing interests.

## Authors’ contributions

GCG performed and analyzed the majority of the experiments and helped design the experimental strategy. CA and XR helped with the data in Figures 1 and 4. JDWC oversaw the whole project and wrote the manuscript together with GCG. All authors read and approved the final manuscript.

## Supplementary Material

Additional file 1: Movie S1 Timelapse movie of a transplanted ectopic cluster of cells labelled with H2B-RFP and Pard3-GFP from 13 to 19 hpf. Separate colour channels and the merged movie are maximum confocal Z-series projections and are shown side-by-side. The nuclei in the cluster are constantly changing place throughout the movie. Pard3-GFP first appears as puncta scattered throughout the cluster, which then coalesce towards the centre over time. Frames are every 5 minutes. Click here for file

Additional file 2: Movie S2 Transverse view timelapse movie of six isochronic cells labelled with H2B-RFP and Pard3-GFP transplanted into one side of the host hindbrain. During the movie, which runs from 15 to 18 hpf, each cell moves towards the midline of the host neural rod where it divides (highlighted by a blue arrow). The medial daughter cell extends to touch the contralateral side of the rod and thus bilateral pairs of cells are generated. Frames are every 5 minutes. White dots indicate the edge of the neural rod, white arrows indicate the position of the midline. Click here for file

Additional file 3: Movie S3 Dorsal view timelapse movie of young cells labelled with H2B-RFP and transplanted into the hindbrain of a host embryo that is labelled with H2B-GFP. Two cell divisions are circled. The young cell (red nucleus) divides with the orientation of a C-division even though the host cell (green nucleus) divides with an orientation of a D-division, indicating that the young cell is dividing with an orientation typical of its age, not the environment. Frames are every 5 minutes. Click here for file

Additional file 4: Figure S1 Pharmacological inhibitors can be used to reversibly block the cell cycle during gastrulation, related to Figure 5. **(A-F)** Maximum projections of control and aphidicolin and hydroxyurea treated (division inhibited) embryos stained for phosphohistone H3 in red to visualize cells undergoing mitosis. All nuclei are labeled in green with sytox-green. (A,B) After 1 h of incubation in aphidicolin and hydroxyurea the number of mitotic figures was greatly reduced in these embryos (n = 6) compared to control embryos (n = 6). (C,D) At the end of the incubation period cell division was still markedly reduced (controls n = 8, division inhibited n = 8). (E,F) At 1 h after wash the number of mitotic figures in division-inhibited embryos remained low (n = 5) compared to control embryos (n = 6). **(G)** Graph showing that cell division is reduced to less than 20% of the wild-type level of cell divisions when embryos are treated with aphidicolin and hydroxyurea and remains reduced for 1 h after wash out of the drugs. Scale bar in A is 100 μm. Click here for file

Additional file 5: Movie S4 Timelapse movie of two cell divisions (blue dots) in a 14th/15th cycle embryo labelled with mem-GFP and H2B-RFP. Both cells divide close to the midline in the medio-lateral axis, and the medial daughter cell crosses the developing midline, to form two pairs of cells. Frames are every 5 minutes. Click here for file
