# Supplementary figures and images for: Developmental time rather than local environment regulates the schedule of epithelial polarization in the zebrafish neural rod
Source: Neural Dev. 2013 Mar 24;8:5. doi: 10.1186/1749-8104-8-5 (PMC3623869; doi:10.1186/1749-8104-8-5)

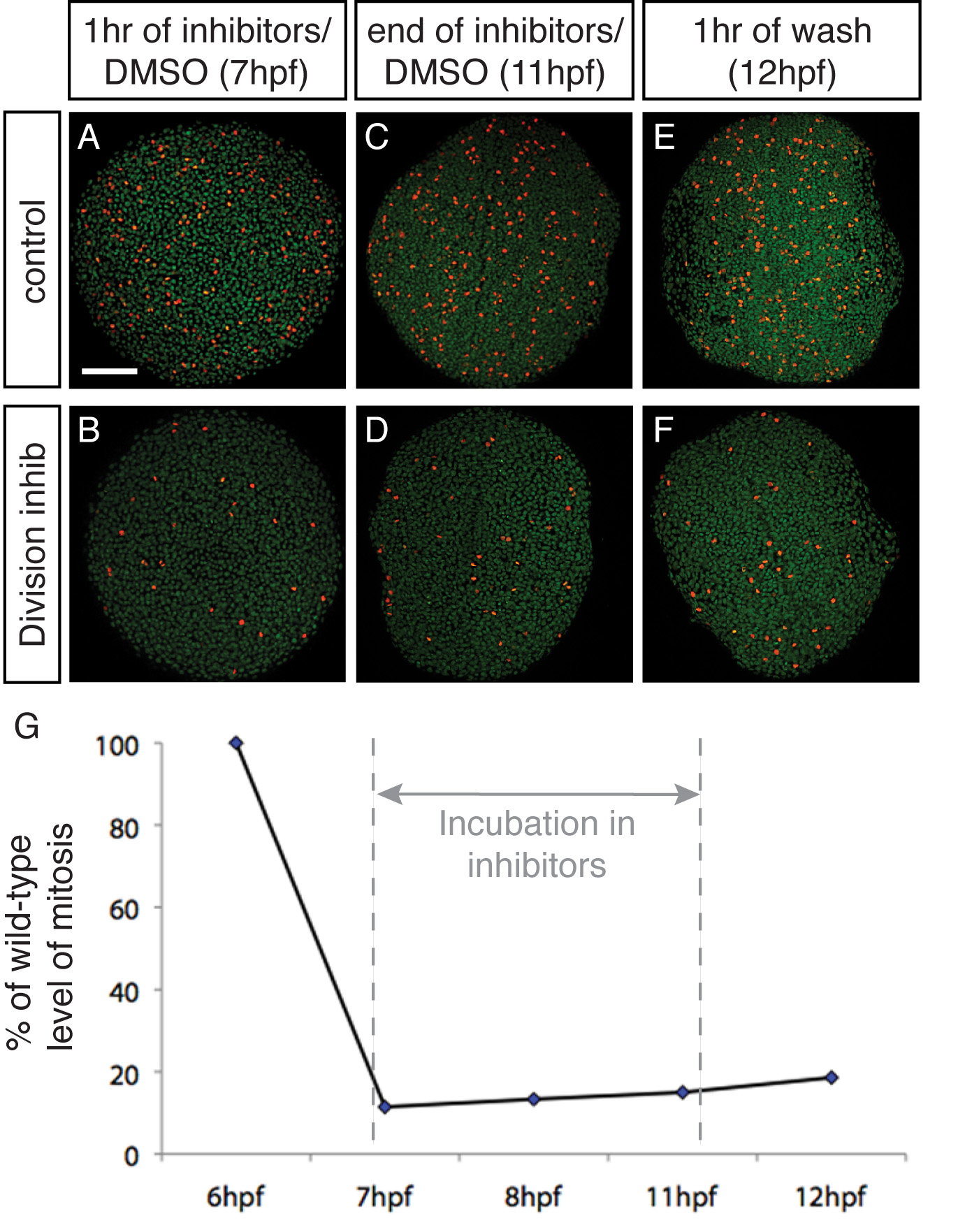

Supplement: Additional file 4: Figure S1 — Pharmacological inhibitors can be used to reversibly block the cell cycle during gastrulation, related to Figure 5. (A-F) Maximum projections of control and aphidicolin and hydroxyurea treated (division inhibited) embryos stained for phosphohistone H3 in red to visualize cells undergoing mitosis. All nuclei are labeled in green with sytox-green. (A,B) After 1 h of incubation in aphidicolin and hydroxyurea the number of mitotic figures was greatly reduced in these embryos (n = 6) compared to control embryos (n = 6). (C,D) At the end of the incubation period cell division was still markedly reduced (controls n = 8, division inhibited n = 8). (E,F) At 1 h after wash the number of mitotic figures in division-inhibited embryos remained low (n = 5) compared to control embryos (n = 6). (G) Graph showing that cell division is reduced to less than 20% of the wild-type level of cell divisions when embryos are treated with aphidicolin and hydroxyurea and remains reduced for 1 h after wash out of the drugs. Scale bar in A is 100 μm. [file 1749-8104-8-5-S4.jpeg]
